# In Response

**DOI:** 10.4269/ajtmh.2011.11-0317b

**Published:** 2011-09-01

**Authors:** Debbie Humphries, Emily Mosites, Joseph Otchere, Richard Bungiro, Hinckley Jones-Sanpei, Lisa Harrison, Michael Wilson, Michael Cappello

**Affiliations:** Yale University School of Medicine; New Haven CT; E-mail: debbie.humphries@yale.edu; Tennessee Department of Health; Nashville TN; Noguchi Memorial Institute for Medical Research; University of Ghana; Accra; Brown University; Providence, RI; University of North Carolina; Chapel Hill NC; Yale University School of Medicine; New Haven CT; Noguchi Memorial Institute for Medical Research; University of Ghana; Accra; Yale University School of Medicine; New Haven CT; E-mail: michael.cappello@yale.edu

Dear Sir:

We appreciate the letter of Montresor and others,^1^ and are grateful for their level of attention to the data reported from this study.^2^ Unfortunately, the concerns raised do not accurately reflect our observations, nor are they warranted based on the paper's stated conclusions.

The writers point out that cure rate is an inadequate measure of drug effectiveness because of its dependence on pretreatment intensity. However, in our study population, we reported that pretreatment intensity did not correlate with cure rate. Moreover, nearly all of the hookworm infection intensities were light, which should have predicted a better response to albendazole. Our data show that this was not the case in the study population.

The writers also suggest that egg reduction rate is a better measure of drug effectiveness than cure rate, a claim we neither refute nor promote in the paper. They state that the fecal egg reduction rate of 81.5% we report “corresponds to the normal range of efficacy of albendazole against this parasite.” Interestingly, however, a recent publication (*PLoS Negl Trop Dis* 2011;5:e948) co-authored by three of the writers (Montresor, Engels, and Albonico) notes that, “… a Fecal Egg Count Reduction (based on arithmetic means) of > 95% for *Ascaris lumbricoides* and > 90% for hookworms should be the expected minimum in all future surveys, and that therapeutic efficacy below this level following a single dose of albendazole should be viewed with concern in light of potential drug resistance.” Applying this standard, it would seem that our results should raise concern about resistance.

The writers fail to comment on what is perhaps the most significant finding in our study, namely that among those subjects who remained positive following treatment with albendazole, there was no statistically significant reduction in fecal egg excretion, and nearly half had higher egg counts on follow-up examination. To address potential mechanisms, we considered a number of possible explanations, including host factors, pretreatment intensity, and inactive drug, none of which were supported by the data. In the absence of alternative explanations, we believe that the possibility of resistance should be considered, especially in light of the implications for current global policies.

Finally, it must be understood that we do not claim in the paper to have determined that the mechanism underlying low cure rates in this population is genetically mediated parasite resistance. Nor do we render any judgment on the relative value of cure rate versus egg reduction rate as a means of monitoring helminth control programs or informing policy. Our colleagues are entitled to their opinion as to how these complementary tests should be implemented, as has been stated in the recently published commentary noted in their letter. Moreover, we wholeheartedly agree that the World Health Organization (WHO) should be concerned about “unjustified claims of drug resistance,” but only when those claims are actually made. Given what has been learned about anthelminthic resistance from the veterinary nematode experience, coupled with the recent commitment of funds to scale up administration of albendazole across sub-Saharan Africa, we would furthermore advocate at the very least that mechanisms to monitor for resistance should be routinely incorporated into deworming programs.

As for the inference that our conclusions suggest a “poor understanding of STH biology and control strategies,” the authors of the manuscript stand by our data and its interpretation, which passed rigorous peer review by *The Journal* and is informed by decades of work in laboratory and field-based research. We welcome future dialogue with colleagues who share our commitment to improving global health through research on endemic infectious diseases.
